# Band keratopathy and conjunctival calcification in end-stage kidney disease: epidemiology, pathophysiology and clinical management

**DOI:** 10.1093/ckj/sfag039

**Published:** 2026-02-12

**Authors:** Nanmei Liu, Ruizhen Ren, Haolin Gou, Maoting Li, Naiying Lan, Fanzhou Zeng, Bo Yang

**Affiliations:** Department of Nephrology, Naval Medical Center of PLA, Naval Medical University, Shanghai, China; Department of Ophthalmology, Dahua Hospital, Shanghai, China; Department of Ophthalmology, Naval Medical Center of PLA, Naval Medical University, Shanghai, China; Department of Nephrology, Naval Medical Center of PLA, Naval Medical University, Shanghai, China; Department of Nephrology, Naval Medical Center of PLA, Naval Medical University, Shanghai, China; Department of Nephrology, Naval Medical Center of PLA, Naval Medical University, Shanghai, China; Department of Nephrology, Naval Medical Center of PLA, Naval Medical University, Shanghai, China

**Keywords:** chronic kidney disease–mineral and bone disorder, conjunctival and corneal calcification, end-stage kidney disease, hyperphosphataemia, metastatic calcification

## Abstract

Conjunctival and corneal calcification, particularly its severe manifestation, band keratopathy, represents a prevalent yet frequently overlooked form of metastatic calcification in end-stage kidney disease (ESKD). This review integrates current evidence on the epidemiology, pathophysiology and interdisciplinary management of uraemic ocular calcification. The pathogenesis is conceptualized through a ‘two-hit’ model: the systemic ‘first hit’ involves the specific milieu of chronic kidney disease–mineral and bone disorder driven by hyperphosphataemia, calcium load, secondary hyperparathyroidism and a critical deficiency in calcification inhibitors such as fetuin-A and Klotho. The ‘second hit’ comprises local ocular triggers, including tear film instability, inflammation and localized alkalosis, which precipitate mineral deposition in the supersaturated environment.

## INTRODUCTION

### Search strategy and selection criteria

We conducted a narrative review of the literature using the PubMed, Embase and Web of Science databases for articles published up to November 2025. Search terms included ‘band keratopathy,’ ‘conjunctival calcification,’ ‘chronic kidney disease,’ ‘end-stage renal disease, (ESRD)’ ‘end-stage kidney disease, (ESKD)’ and ‘dialysis’. We prioritized data from large-scale epidemiological cohorts and prospective studies utilizing high-sensitivity imaging [e.g. anterior-segment optical coherence tomography (AS-OCT)]. Case reports and smaller series were included specifically to illustrate rare clinical presentations or novel therapeutic interventions. Evidence was synthesized to distinguish between robust epidemiological associations and hypothesized pathophysiological mechanisms.

### Metastatic calcification in ESKD: a systemic process with local manifestation

In patients with advanced chronic kidney disease (CKD) and ESKD, the failure of renal excretion leads to the systemic retention of minerals, most notably phosphate, and a cascade of hormonal derangements [1, 2]. This environment, supersaturated with calcium and phosphate, provides the foundation for metastatic calcification—the deposition of calcium phosphate salts into previously normal, uninjured soft tissues. This process is distinct from dystrophic calcification, which occurs in tissues that are already damaged or necrotic.

In the uraemic state, the eye is recognized as one of the primary and frequent targets for metastatic calcification [3]. This is not an isolated event; the same systemic drivers are concurrently promoting calcification in other soft tissues, including blood vessels, heart valves, skin and periarticular structures [4, 5].

The development of ocular calcification can be conceptualized through a ‘two-hit’ hypothesis. The ‘first hit’ is the systemic, pro-calcific state of uraemia, defined by the failure of renal excretion and the resulting supersaturation of calcium and phosphate in the blood [6]. The ‘second hit’ is a local ocular trigger; factors within the eye’s microenvironment, such as local pH changes, inflammation or tear film instability, create a nidus that initiates the precipitation of these supersaturated minerals out of solution and into the ocular tissues [7].

### Histopathology: calcium phosphate deposition in ocular tissues

The specific location of the mineral deposits defines the clinical manifestation. Calcific band keratopathy (CBK) is characterized by the deposition of calcium phosphate, chemically identified as hydroxyapatite crystals. Histopathologically, these deposits are found in the most anterior layers of the cornea. The deposition specifically involves the epithelial basement membrane [8, 9], Bowman’s layer and the superficial anterior stroma. As these deposits accumulate, they form an opacifying layer that can obstruct vision [10, 11]. In the conjunctiva, which is often the first ocular tissue to show calcification, the deposits are typically found in the basal lamina of the epithelium and within the subepithelial tissues. This process is often associated with a concurrent degeneration of the subepithelial elastic tissue [12].

### Clinical presentation and symptomatology

The clinical presentation of uraemic ocular calcification exists on a spectrum, from being entirely asymptomatic to causing severe, vision-threatening discomfort [13]. Many patients, particularly in the early stages, are asymptomatic. The calcific deposits are discovered incidentally during a slit lamp examination as small, punctate, chalky or white plaques in the conjunctiva, often near the limbus.

As the condition progresses, symptoms emerge (Box 1).

Box 1.

- Visual disturbance: As corneal deposits (band keratopathy) coalesce and advance from the periphery toward the centre, they enter the visual axis, causing significant blurred vision.
- Ocular discomfort: Patients frequently report a foreign body sensation, often described as feeling ‘sand’ or ‘grit’ in the eye. This is accompanied by general eye irritation and redness.
- Pain: If the overlying corneal epithelium becomes unstable and breaks down over the rough, elevated calcific plaques, patients can experience recurrent corneal erosions, which are acutely painful.
- Photophobia: A significant intolerance to light is a common complaint [14].

A specific and important clinical sign is the ‘red eye of uraemia’ or ‘red eye of renal failure’ [15]. This entity is not simply the presence of calcification but rather an acute inflammatory reaction to it. It is characterized by painful irritation and a distinct ‘waxy red, more or less diffuse, episcleral and conjunctival hyperaemia’ [16]. This reaction is thought to be triggered by the deposition of microcrystals or subsequent erosion of the epithelium. This ‘red eye’ is often transient, lasting for a few days, and may recur as new calcium deposition occurs. Its appearance is a critical clinical indicator of an active, ongoing metabolic process and has been correlated with high serum calcium levels.

### Diagnostic evaluation: from slit lamp to AS-OCT

Diagnosis is primarily clinical (Table 1), relying on a thorough ophthalmic examination [17]. Slit lamp biomicroscopy remains the gold standard for clinical diagnosis. The deposits appear as a characteristic band-shaped, horizontal, grey-white, subepithelial opacity. It is most prominent in the interpalpebral fissure (the part of the eye exposed when the eyelids are open). The band classically begins at the 3 and 9 o’clock positions at the limbus (the border of the cornea and sclera) and slowly progresses centrally [8, 13]. A key diagnostic feature is the ‘cheesy’ appearance of the plaque, which is often punctuated by small, clear ‘holes’. These clear spots are pathognomonic, representing areas where corneal nerves pass through Bowman’s layer, inhibiting the local deposition of crystals [18]. Conjunctival calcification appears as small, discrete, chalky white deposits, typically located in the interpalpebral conjunctiva near the limbus [19].

**Table 1:** Diagnostic grading systems for uraemic ocular calcification.

Grading system	Methodology	Grade/score description	Clinical utility
Porter & Crombie/Tokuyama [3]	Slit lamp (visual inspection)	Grade 0: normal (no deposits)Grade 1: conjunctival deposits onlyGrade 2: conjunctival + strictly limbal depositsGrade 3: conjunctival + single line of corneal depositsGrade 4: clear single line of corneal deposits + conjunctival depositsGrade 5: more extensive corneal deposits + conjunctival deposits	Standard clinical gradingUsed in major prognostic studies (e.g. Hsiao *et al*. mortality study)Limitations: subjective; misses subclinical deposits
AS-OCT Grading (Pessoa *et al*.) [22]	AS-OCT (tomographic imaging)	Grade 0: no hyperreflective signalsGrade 1: isolated conjunctival depositsGrade 2: linear conjunctival depositsGrade 3: clumped/nodular conjunctival depositsGrade 4: corneal involvement (band keratopathy)	High sensitivity: detects subclinical calcification missed by slit lampObjective: quantifies depth and density (shadowing)Monitoring: best for tracking regression post-treatment (e.g. parathyroidectomy

AS-OCT is an emerging non-invasive imaging modality that provides high-resolution, cross-sectional images of the anterior eye [20]. It represents a significant technological advance in this area. AS-OCT can detect calcific deposits before they become apparent on a slit lamp examination. The deposits are identified as hyperreflective signals that cast a ‘posterior acoustic shadow’, a definitive sign of a dense, light-blocking deposit [21, 22]. This technology allows for a new, more objective classification and scoring of conjunctival and corneal calcification (CCC). Furthermore, AS-OCT has been used to document the reversal and reduction in size of ocular calcifications following systemic treatment (i.e. parathyroidectomy), demonstrating its potential as a tool for monitoring therapeutic response [21].

## EPIDEMIOLOGICAL BURDEN OF BAND KERATOPATHY AND CONJUNCTIVAL CALCIFICATION IN CKD

### Global prevalence of CKD and associated ocular complications

CKD is a global public health crisis, with systematic reviews estimating a global prevalence of 14.2% for stages 1–5 [23]. As the world’s population ages, the burden of CKD and its myriad complications, including chronic kidney disease–mineral and bone disorder (CKD-MBD) and associated ocular diseases, is expected to increase. While CKD is associated with a wide array of ocular problems—including diabetic/hypertensive retinopathy, glaucoma and cataracts [24, 25]—calcific keratopathy and conjunctival calcification are unique in that they are a direct metabolic consequence of the uraemic state itself.

### Incidence and prevalence of ocular calcification in dialysis cohorts

The prevalence of CCC in the maintenance dialysis population is exceptionally high, although reported figures vary widely depending on the sensitivity of the diagnostic method employed. Clinical studies have reported prevalence rates of 43.8% [26] and 60.6% [27] in haemodialysis (HD) cohorts. However, when highly sensitive AS-OCT is used for screening, the prevalence of CCC has been reported to be as high as 82.7% [22]. This suggests that a vast majority of dialysis patients harbour some degree of subclinical ocular calcification (Table 2).

**Table 2:** Aggregated epidemiological data on ocular calcification in CKD/ESKD.

Study	Study design and population	Diagnostic method	Key findings (incidence/prevalence/risk)
Weng *et al*. (2016) [8]	Retrospective cohort (94 039 ESKD versus 94 039 controls)	ICD-9 codes (clinical diagnosis)	Incidence rate ratio (IRR): 12.21 (*P* < .0001)Adjusted HR: 11.56Age paradox: incidence highest in age <50 years (8.37/10 000 PY) versus age >65 years (1.86/10 000 PY)
Hsiao *et al*. (2011) [17]	Prospective cohort (109 maintenance HD patients)	Slit lamp photos (Porter–Crombie grade)	Prevalence: 39.4% (mild), 32.1% (moderate), 28.4% (severe)
			Mortality: severe CCC is an independent predictor of 1-year all-cause mortality (HR 1.26 per score point)
Pessoa *et al*. (2023) [22]	Cross-sectional (29 maintenance dialysis patients)	AS-OCT (high sensitivity)	Prevalence: 82.7% (very high)Correlation: no significant correlation found between AS-OCT calcification score and coronary artery calcium (Agatston) score
Tokuyama *et al*. (2002) [3]	Cross-sectional (44 maintenance HD patients)	Slit lamp	Prevalence: 79.5%Correlation: strong correlation with dialysis vintage, serum Ca, P and Ca × P product
Ahuja *et al*. (2022) [62]	Comparative (76 HD versus 32 PD patients)	Impression cytology	Prevalence: HD (0%) versus PD (6.3%)Difference not statistically significant (*P* = .09). Suggests modality may not be primary driver.

PY: person-years.

### Comparative risk analysis: ESKD versus the general population

The most robust data quantifying the specific risk of band keratopathy comes from a large-scale, retrospective, nationwide matched cohort study in Taiwan [8]. When comparing >90 000 ESKD patients with matched controls, the study established that dialysis patients have a >11-fold increased risk [adjusted hazard ratio (HR) 11.56] of developing this corneal complication (Table 2). Interestingly, the study revealed an inverse relationship between age and incidence. The risk amplification was highest in the youngest cohort (<50 years) and lowest in the elderly (>65 years). This ‘age paradox’ strongly suggests a survivorship bias: younger patients who develop band keratopathy likely represent a phenotype with aggressive systemic calcification [28]. These patients may suffer early cardiovascular mortality, leaving a survivor cohort of elderly patients with less aggressive mineral dysregulation. Thus band keratopathy in a young patient should be viewed as a critical biomarker of a lethal systemic process.

## AETIOLOGY AND RISK FACTORS: A MULTIFACTORIAL MODEL

**Figure 1:**
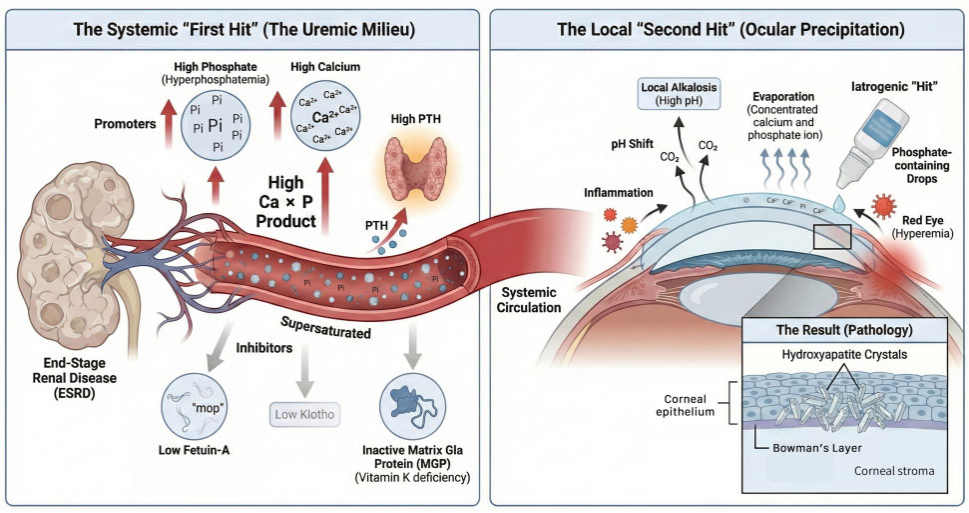
The ‘two-hit’ pathophysiology of uraemic ocular calcification. Pi: inorganic phosphate.

The development of uraemic ocular calcification is not due to a single factor but rather the result of systemic, local and iatrogenic factors converging on the ocular surface (Figure 1).

### Systemic comorbidities

Beyond the state of ESKD itself (the primary risk factor), several systemic comorbidities are independently associated with an increased risk.

#### Diabetes mellitus (DM)

DM is a significant independent risk factor for developing band keratopathy [adjusted odds ratio (OR) 2.617] [29]. This highlights a ‘multihit’ process. DM is known to promote vascular calcification on its own through mechanisms of oxidative stress and chronic inflammation. When the pro-inflammatory state of diabetes is combined with the pro-calcific state of CKD-MBD, the result is a synergistic and accelerated deposition of minerals in soft tissues [30, 31].

#### Hypertension

A common comorbidity in CKD, hypertension has also been shown to be associated with the severity of CCC scores in dialysis patients [17].

### Local ocular risk factors: the precipitation trigger

Systemic supersaturation (hit 1) is necessary, but local factors (hit 2) often determine where and when precipitation occurs. Chronic ocular inflammation states are potent independent risk factors. Patients with this form of chronic iridocyclitis have a dramatically increased risk of band keratopathy (adjusted OR 9.5 [29] or 4.3 [8]). Inflammation likely alters local tissue pH, vascular permeability and cellular function, creating a nidus for calcification [18]. Phthisis bulbi—a shrunken, non-functional eye, often the end-result of severe trauma or inflammation—carries an astronomical adjusted OR of 141.9 for band keratopathy [29]. This finding, while an extreme case, powerfully demonstrates that profound local tissue disorganization and chronic inflammation are an overwhelming attractant for calcium precipitation in a patient who is systemically supersaturated.

Altered local pH and tear dynamics also contribute. The classic theory explaining the interpalpebral location (the exposed part of the eye) of band keratopathy and CCC is local alkalinity. It is hypothesized that carbon dioxide diffuses from the exposed ocular surface into the air, causing a localized increase in pH [32]. In a patient with high serum calcium, the tear film is also calcium-rich [9]. This slight shift toward alkalinity is just enough to exceed the calcium × phosphate (Ca × P) solubility product, triggering precipitation. This effect is exacerbated by keratitis sicca, which is very common in dialysis patients [19]. Tear evaporation concentrates the tear film, further increasing the concentration of calcium and phosphate and promoting precipitation [16].

Iatrogenic risk represents a critical, modifiable and often-overlooked iatrogenic risk factor. Dialysis patients frequently suffer from dry, red, irritated eyes and ocular hypertension, leading to the chronic use of topical eye drops (e.g. artificial tears, glaucoma medications). Many of these common formulations use phosphate as a buffer or preservative [33]. In a normal patient, this is harmless. However, in a uraemic patient with high tear calcium levels, the addition of a phosphate-loaded eye drop creates a massive, localized phosphate burden [8]. This combination is the perfect chemical recipe for acute and rapid calcific band keratopathy. This iatrogenic calcification can be severe, dense and involve deeper corneal layers, sometimes necessitating surgical keratoplasty (corneal transplant) [34]. This scenario highlights a dangerous ‘interdisciplinary blind spot’: the nephrologist works to lower systemic phosphate while the ophthalmologist, perhaps unaware of the systemic metabolic context, may prescribe a topical medication that creates a localized phosphate overload, directly causing the complication. The clear clinical mandate is that ophthalmologists should be acutely aware of this risk and preferentially prescribe phosphate-free formulations for all CKD and dialysis patients.

## THE CENTRAL ROLE OF CKD-MBD

### The drivers: calcium load, phosphate and parathyroid hormone (PTH)

The triad of calcium load, hyperphosphataemia and secondary hyperparathyroidism (SHPT) constitutes the fundamental engine of metastatic calcification in ESKD [35].

Although the incidence of overt, severe hypercalcaemia has decreased with the advent of calcimimetics and low-calcium dialysate, the ‘calcium load’ remains a primary driver of ocular calcification. Contrary to the assumption that hypercalcaemia is no longer relevant, clinical studies have established a robust statistical link. In a prospective cohort study by Hsiao *et al*. [17], corrected serum calcium was identified as a significant independent risk factor for the severity of CCC and mortality (*P* < .05). Similarly, Tokuyama *et al*. [3] demonstrated a direct positive correlation between serum calcium levels and the prevalence of limbal calcification. Furthermore, Weng *et al*. [8] validated that patients with higher Ca × P products had significantly higher risks of band keratopathy. Mechanistically, in the setting of inhibitor deficiency, even ‘high-normal’ serum calcium levels can exceed the precipitation threshold in ocular tissues.

#### Hyperphosphataemia

As glomerular filtration rate falls, phosphate excretion fails, leading to hyperphosphataemia [36]. This is a key independent risk factor for CCC [37]. It correlates with the severity of CCC and, beyond simply precipitating, also acts as a signalling molecule that can induce a phenotypic transformation of vascular and corneal cells into osteochondrogenic cells [38].

#### PTH

SHPT is an almost universal complication of advanced, untreated CKD. PTH is not a passive bystander, it is an active driver [39]. Multiple studies have found a direct, positive correlation between serum PTH levels and the severity of CCC [3, 40, 41]. One study found that patients in the moderate/severe CCC group had significantly higher mean PTH levels (357.3 pg/ml) than those in the mild group (228.4 pg/ml) [26]. The research also found a positive association between CCC scores and PTH levels (*R*^2^ = 0.434). This is because high PTH levels drive high bone turnover, continuously mobilizing calcium and phosphate from the skeletal reservoir into the bloodstream. This action directly ‘feeds’ the high Ca × P product, promoting precipitation.

### The defective defence: deficiency of calcification inhibitors

The modern understanding of uraemic calcification has shifted. It is no longer seen as just a passive process driven by an excess of ‘promoters’ (calcium and phosphate). It is now understood to be an active, cell-mediated process resulting from the catastrophic failure of the body’s native ‘inhibitor’ systems [42]. CKD functions as a ‘calcification inhibitor–deficient’ state. While direct ocular tissue staining for these proteins is rare, their systemic deficiency provides the mechanistic explanation for why these patients precipitate calcium even at non-extreme serum levels.

#### The FGF-23/Klotho axis

In healthy physiology, fibroblast growth factor 23 (FGF23) is a hormone that tells the kidney to excrete phosphate [43, 44]. As CKD progresses, FGF23 levels rise exponentially in a futile attempt to maintain phosphate balance [45]. Klotho, a protein produced primarily by the kidney, is the essential co-receptor that allows FGF23 to work [46]. In ESKD, Klotho production plummets [47]. This creates a dual-problem state: Klotho deficiency and massive FGF23 resistance. The resulting sky-high FGF23 levels, now unmoored from their co-receptor, are thought to have ‘off-target’, Klotho-independent toxic effects, directly promoting cardiovascular damage [48].

#### Fetuin-A and the systemic buffer

Fetuin-A is a potent, circulating glycoprotein that acts as a systemic ‘mop’. It is one of the most important inhibitors of calcification, binding excess calcium and phosphate in the blood to form soluble ‘calciprotein particles’, preventing them from precipitating [49]. In CKD and dialysis patients, serum fetuin-A levels are significantly reduced [50]. Low fetuin-A levels are strongly associated with increased vascular calcification and mortality [51]. It is hypothesized that this loss of systemic buffering capacity lowers the solubility threshold in the ocular vasculature and tear film.

#### Matrix Gla protein (MGP)

This is a powerful local inhibitor that works within the tissue wall to prevent calcification [52]. MGP is vitamin K-dependent; it must be ‘activated’ by a process called carboxylation [53]. Many CKD patients are functionally vitamin K deficient, leaving their MGP inactive [54]. An MGP-deficient state (as seen in knockout mice) leads to massive and rapid vascular calcification [55, 56].

## IMPACT OF RENAL REPLACEMENT THERAPY ON OCULAR CALCIFICATION

### Dialysis vintage: the critical role of cumulative exposure

A consistent finding across numerous studies is the critical role of ‘dialysis vintage’, or the total duration of time a patient has been on renal replacement therapy [26, 57, 58]. There is a significant, positive correlation between the number of years on dialysis and both the prevalence and severity of CCC [17]. This strongly implies a cumulative dose–response relationship. Ocular calcification is not an acute event but rather the slow, progressive result of years—or even decades—of cumulative exposure to the uraemic milieu and the imperfect metabolic correction provided by dialysis [59].

### Dialysis modality and adequacy

A logical question is whether the type of dialysis affects the risk. While HD involves rapid osmotic shifts compared with the stability of peritoneal dialysis (PD) [24, 60, 61], the evidence for a difference in calcification risk is inconclusive. One 2022 study [62] found no cases of conjunctival calcification in the HD group, but a 6.3% prevalence in the PD group. This difference was not statistically significant (*P* = .09). Another study that included both 44 PD and 19 HD patients pooled their data for analysis, preventing a direct comparison of risk [37]. Similarly, studies on coronary calcification progression found no significant difference between HD and PD patients. This suggests that the modality itself is likely less important than the overall quality of metabolic control achieved [61].

Regarding adequacy, standard Kt/V measures do not correlate with calcification severity [17]. However, the dialysate calcium concentration is a critical, actionable parameter. Historically high dialysate calcium [63] (e.g. 1.75 mmol/l) created a positive calcium influx during treatment [64]. Evidence now supports lowering dialysate calcium (e.g. 1.25 mmol/l) [65] to attenuate calcification progression, a strategy that must be individualized to the patient’s oral calcium load.

## THERAPEUTIC MANAGEMENT: A DUAL-PRONGED APPROACH

**Figure 2:**
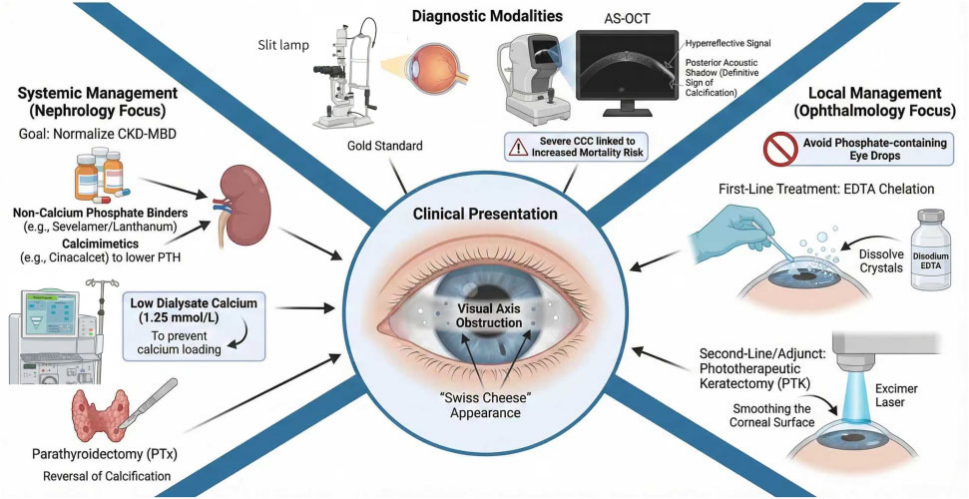
Integrated multidisciplinary management strategy.

Management of uraemic ocular calcification is two-fold: first, aggressive systemic treatment to control the underlying metabolic derangement and prevent progression and, second, local ocular treatment to relieve symptoms and restore vision (Figure 2).

### Systemic control: aggressive CKD-MBD management

While no large randomized controlled trials have used ocular calcification as a primary endpoint for phosphate binders or calcimimetics, systemic management follows the established guidelines for preventing metastatic calcification in ESKD. Phosphate control is the cornerstone, typically achieved through dietary restriction and the use of phosphate binders [66]. A critical management decision involves the choice of binder. While calcium-based binders (e.g. calcium carbonate) are effective, they contribute to the patient’s total calcium load, which can paradoxically worsen calcification [67]. Consequently, non-calcium-based binders such as sevelamer or lanthanum are often preferred, as they control phosphate levels without increasing the calcium load.

Control of SHPT is equally vital. Vitamin D analogues (e.g. calcitriol) are commonly used to suppress PTH secretion, but they carry the side effect of increasing both serum calcium and phosphate absorption, which can exacerbate the Ca × P product [68]. Calcimimetics, such as cinacalcet, represent a major therapeutic advance in this regard. By binding to the calcium-sensing receptor on the parathyroid gland and ‘mimicking’ calcium, these agents suppress PTH secretion without raising serum calcium or phosphate levels [69]. For patients with severe, refractory SHPT unresponsive to medical therapy, parathyroidectomy (PTx) remains the definitive treatment [70]. Proof of concept for the reversibility of these lesions was demonstrated in a 2020 case report utilizing AS-OCT, which showed significant resorption of ocular calcifications just 6 months after PTx [21].

### Local ocular intervention: symptomatic relief

When band keratopathy progresses to cause pain, recurrent erosions or vision loss, local treatment is indicated [71] (Table 3). Topical disodium ethylenediaminetetraacetic acid (EDTA) chelation is the first-line, safest and most common treatment for symptomatic band keratopathy [72]. The procedure involves removing the corneal epithelium, often with a 20% alcohol solution, followed by the application of a disodium EDTA solution (typically 0.37–1%) to the cornea. EDTA acts as a chelating agent, binding the insoluble calcium in the plaque to form a soluble calcium–EDTA complex, softening the deposit for easy removal. This method is highly effective, with a success rate of 97.8% in clearing the visual axis and visual acuity maintenance or improvement in 79.8% of patients. While the recurrence rate is moderate (28.1%), the need for retreatment is low, making it a favourable option.

**Table 3:** Comparative efficacy of therapeutic interventions for band keratopathy.

Intervention	Mechanism	Success rate (visual axis clearance/acuity)	Recurrence rate	Key advantages/disadvantages
EDTA chelation	Chemical chelation of calcium	97.8% visual axis clearance79.8% visual axis improved/stable	28.1% (moderate)Only 4.5% needed retreatment	Pros: low cost, can be done at slit lamp, minimally invasiveCons: manual scraping required; incomplete removal of deep deposits
PTK (excimer laser)	Laser ablation of tissue/calcium	High efficacy for smoothing irregular surfaces	Rare/delayed (significantly lower than EDTA)	Pros: extremely precise, leaves smooth surface, treats deeper stromal depositsCons: expensive, refractive shift (hyperopia), risk of haze
Superficial keratectomy	Manual scraping (blade/burr)	Variable; dependent on surgeon skill	Variable	Pros: simple, no special chemicalsCons: risk of irregular astigmatism, scarring, incomplete removal
Parathyroidectomy	Systemic removal of PTH driver	Reversal of calcification size/number observed at 6 months	Not applicable (systemic cure)	Pros: treats the root cause; benefits bone/heartCons: major surgery; risks of hypocalcaemia/hungry bone syndrome

For denser or more irregular plaques, simple EDTA chelation may be supplemented with manual superficial keratectomy or phototherapeutic keratectomy (PTK). PTK utilizes an excimer laser to ablate the superficial cornea and embedded calcium deposits [73]. This technique offers high precision and a smooth postoperative surface, making it preferable for deposits that cause significant surface irregularity or are located in deeper corneal layers [71].

## PROGNOSTIC SIGNIFICANCE: AN OCULAR WINDOW TO SYSTEMIC DISEASE?

### The eye–heart link: medial versus intimal calcification

Historically, CCC has been viewed as a ‘window’ to systemic vascular calcification. Earlier studies supported this, finding correlations between ocular deposits and aortic arch or abdominal aortic calcification [17, 37]. However, recent high-sensitivity imaging has introduced complexity to this relationship. A 2023 study utilizing AS-OCT and cardiac computed tomography found no significant correlation between coronary artery calcium scores and ocular calcification scores [22]. This discrepancy likely stems from distinct pathophysiological pathways. CCC and aortic medial calcification represent ‘pure’ metastatic calcification driven by CKD-MBD (high Ca × P and PTH). In contrast, coronary calcification is often intimal and atherosclerotic, driven by lipids and inflammation. Therefore, while the eye may not perfectly predict coronary atherosclerosis, it remains a valuable clinical sensor for the MBD-driven, medial calcification pathway associated with vascular stiffness and heart failure.

### Ocular calcification as an independent predictor of mortality

The prognostic value of CCC extends beyond mere correlation with other calcific deposits. Findings of Hsiao *et al*. [17] suggest that elevated CCC from an ocular finding is a critical biomarker for mortality risk. In the 109 maintenance HD patients included, severe CCC was identified as a significant independent risk factor for all-cause 1-year mortality, alongside age and corrected serum calcium. The analysis revealed that each 1-point increment in the CCC severity score was associated with a 26.4% increased risk for all-cause mortality, suggesting that a simple slit lamp examination could be a powerful tool for risk stratification.

## FUTURE RESEARCH DIRECTIONS

### Gaps in knowledge and current controversies

Despite significant progress, several critical questions remain unanswered. A primary area of need is the revalidation of prognostic markers. The conflicting findings of Hsiao *et al*. [17] and Pessoa *et al*. [22] highlight the necessity for large, prospective studies using modern, high-sensitivity AS-OCT to determine if subclinical ocular calcification carries the same mortality risk as visible, end-stage disease. Additionally, the impact of dialysis modality remains unclear, with conflicting data on whether PD confers a different risk profile than HD. Research must also pivot from prevention to regression, investigating whether medical management alone can achieve the reversal of calcification seen after PTx. Finally, the paediatric population remains significantly understudied, representing a major gap in the literature.

### Novel diagnostic tools for early detection

Technological advancements offer new avenues for early detection. AS-OCT requires validation in large cohorts to standardize its use as a screening and prognostic tool. Concurrently, retinal imaging using three-dimensional retinal scans (retinal OCT) could provide a parallel assessment of the microvasculature, distinguishing vascular, atherosclerotic complications from metastatic, calcific ones [74]. Furthermore, the development of novel bisphosphonate radiotracers for positron emission tomography imaging may allow for precise systemic quantification of ectopic calcification, enabling robust correlation studies with ocular findings [75].

### Emerging systemic therapeutic targets

Future therapies are moving beyond simple mineral control to target the underlying mechanisms of calcification. Sodium thiosulfate [76–78], currently a rescue therapy for calciphylaxis, is being explored for its calcium-chelating and antioxidant properties in broader systemic and topical applications. Restoring the body’s natural inhibitors is another promising frontier. Strategies include vitamin K supplementation to activate MGP [79], which acts as a natural calcification inhibitor. Future interventions may also involve fetuin-A replacement or synthetic Klotho mimetics to restore the systemic defence against precipitation.

## CONCLUSION

Uraemic ocular calcification is not merely an ophthalmological curiosity but a visible manifestation of a potentially lethal systemic metabolic disease. Its presence should serve as an immediate alarm bell for clinicians, signalling a failure of metabolic control and a heightened risk of mortality. Effective management demands a paradigm shift toward aggressive optimization of CKD-MBD parameters and, crucially, a collaborative partnership between nephrologists and ophthalmologists.

## Data Availability

No new data were created or analysed in this study.
